# Supplementary material for: Measuring inter-protein pairwise interaction energies from a single native mass spectrum by double-mutant cycle analysis
Source: Nat Commun. 2017 Aug 9;8:212. doi: 10.1038/s41467-017-00285-1 (PMC5550451; doi:10.1038/s41467-017-00285-1)
Supplement: Supplementary file 1 — Supplementary Information [file 41467_2017_285_MOESM1_ESM.pdf]

File Name: Supplementary Information
Description: Supplementary Figures, Supplementary Tables and Supplementary Notes

File Name: Peer Review File
Description:

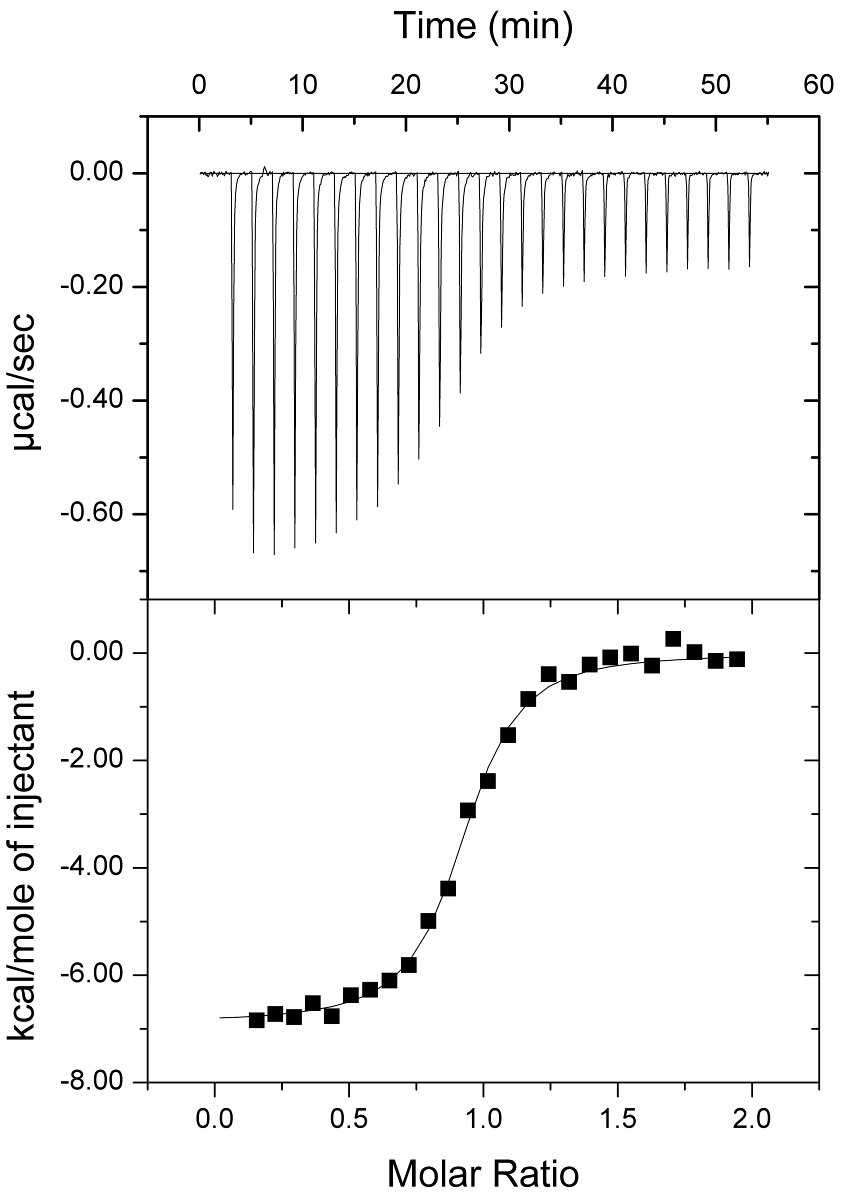

Supplementary Figure 1. **Isothermal calorimetric titration of the S84A mutant of E9 endonuclease by the wild-type bacterial immunity protein, Im2**. The titration was carried out in 250 mM ethylenediammonium diacetate buffer (pH 7.0) at 25 °C as described under Methods. These results show that adding the mutations H131A and R132A to the flexible tail of the E9 S84A mutant has no effect on its binding to wild-type Im2 (see Table 1).

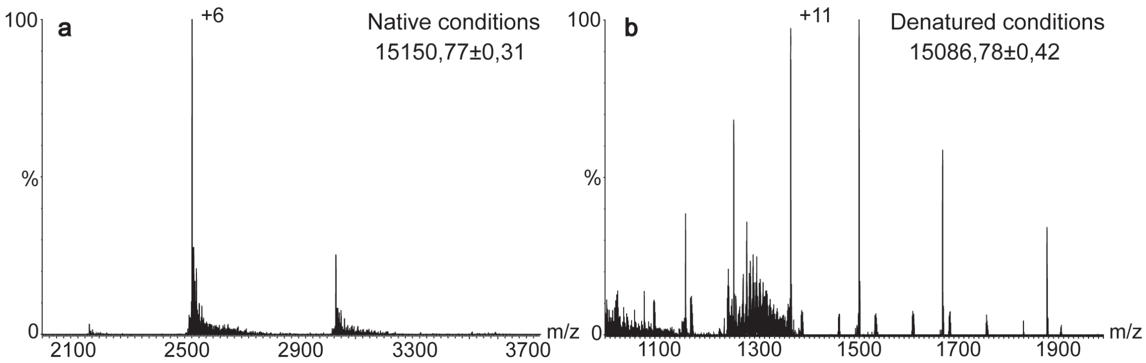

Supplementary Figure 2. An ethylenediammonium ion is bound to the intact E9 protein. (**a**) The determined mass of E9 extracted from data measured under native conditions indicates a 62 Da shift from the expected calculated mass (15,088 Da). This result suggests that an ethylenediammonium ion is associated with the protein. (**b**) A spectrum acquired under denaturing conditions, which disrupts the quaternary structure of the protein, confirms that ethylenediammonium is non-covalently associated with E9.

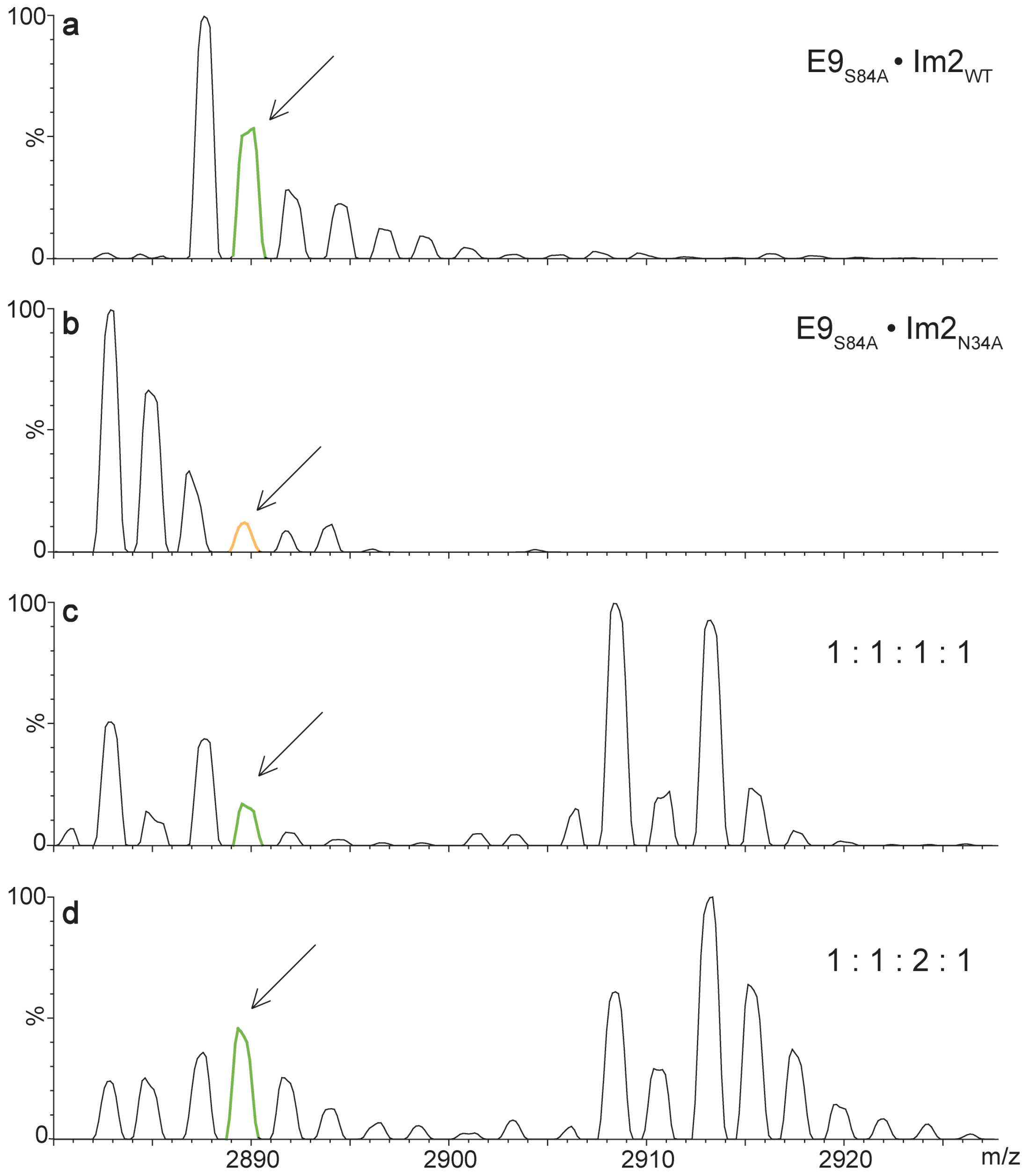

Supplementary Figure 3. Strategy for assigning overlapping peaks exemplified for the peak at 2889 m/z. The peak at 2889 m/z (designated by the arrows) can correspond to the complex of (**a**) E9$_{S84A}$ with wild-type Im2 or (**b**) E9$_{S84A}$ with Im2$_{N34A}$ but not to the complexes of wild-type E9 with Im2$_{N34A}$ or wild-type Im2 (Fig. 3). In order to distinguish between these two possibilities, we compared the spectrum for (**c**) equal concentrations of the four proteins (designated by 1:1:1:1) with one (**d**) when the concentration of wild-type Im2 was increased by two-fold (1:1:2:1). An increase in the relative concentration of wild-type Im2 led to an increase in the peak intensity at 2889 m/z, thereby indicating that this peak corresponds to the complex of E9$_{S84A}$ with wild-type Im2.

Supplementary Note 1

Supplementary Table 1: Concentration ratios of different complexes of wild-type E9 with mutant and wild-type Im2 correspond to the peak area ratios.

**Concentration ratios $Im2_{WT}$: $Im2_{N34A}$: $E9_{WT}$**	**Peak area ratios $Im2_{WT}$•$E9_{WT}$ / $Im2_{N34A}$•$E9_{WT}$**	
	**Charge state +8**	**Charge state +9**
**2:1:164**	2.01 ± 0.13	2.05 ± 0.12
**1:1:82**	1.04 ± 0.05	1.01 ± 0.04
**1:2:164**	0.51 ± 0.02	0.53 ± 0.01

Supplementary Table 2: Concentration ratios of different complexes of wild-type Im2 with mutant and wild-type E9 correspond to the peak area ratios,

**Concentration ratios $E9_{WT}$: $E9_{S84A}$: $Im2_{WT}$**	**Peak area ratios $E9_{WT}$•$Im2_{WT}$ / $E9_{S84A}$•$Im2_{WT}$**	
	**Charge state +8**	**Charge state +9**
**2:1:164**	2.06 ± 0.15	2.04 ± 0.06
**1:1:82**	0.98 ± 0.08	1.03 ± 0.05
**1:2:164**	0.52 ± 0.08	0.53 ± 0.07

The intensities of given concentrations of wild-type and mutant variants of one of the proteins, say E9, were compared in the presence of an excess of a binding partner, say wild-type Im2. Under such conditions, the mutant and wild-type variants of E9 are fully bound to Im2. The results show that the ratio of intensities of the complexes correspond to the ratio of their concentrations, thereby indicating that the ionization efficiencies of the complexes are the same.
